# Kinetics and Mechanisms of the Chromium(III) Reactions with 2,4- and 2,5-Dihydroxybenzoic Acids in Weak Acidic Aqueous Solutions

**DOI:** 10.1155/2010/832768

**Published:** 2010-07-11

**Authors:** Kimon Zavitsanos, Athinoula L. Petrou

**Affiliations:** Laboratory of Inorganic Chemistry, Department of Chemistry, University of Athens, Panepistimioupolis, 15771 Athens, Greece

## Abstract

The reactions of 2,4- and 2,5-dihydroxybenzoic acids (dihydroxybenzoic acid, DHBA) with chromium(III) in weak acidic aqueous solutions have been shown to take place in at least two stages. The first stage of the reactions has an observed rate constant *k*_1(obs)_ = *k*_1_[DHBA] + *C* and the corresponding activation parameters are ΔH_1(2,4)_^≠^ = 49, 5 kJ/mol^−1^, Δ*S*_1(2,4)_^≠^ = −103, 7 J mol^−1^ K^−1^, ΔH_1(2,5)_^≠^ = 60, 3 kJ/mol^−1^, and Δ*S*_1(2,5)_^≠^ = −68, 0 J mol^−1^ K^−1^. These are composite activation parameters and the breaking of the strong intramolecular hydrogen bonding in the two ligands is suggested to be the first step of the (composite) first stage of the reactions. The second stage is ligand concentration independent and is thus attributed to a chelation process. The corresponding activation parameters are ΔH_2(2,4)_^≠^ = 45, 13 kJ/mol^−1^, Δ*S*_2(2,4)_^≠^ = −185, 9 J mol^−1^ K^−1^, ΔH_2(2,5)_^≠^ = 54, 55 kJ/mol^−1^, and Δ*S*_2(2,5)_^≠^ = −154, 8 J mol^−1^ K^−1^. The activation parameters support an associative mechanism for the second stage of the reactions. The various substitution processes are accompanied by proton release, resulting in pH decrease.

## 1. Introduction

Chromium at low concentrations has been proved to be beneficial to many plant species, being toxic in the same plants at higher concentrations [1]. The bioinorganic chemistry of chromium(III) and its biological role is not very well studied because (a) chromium(III) complexes are substitution inert and this implies that a catalytic role is forbidden, (b) chromium(III) complexes are generally redox inactive especially with most of the biological ligands, (c) the electronic spectra of the chromium(III) complexes usually lack of intense characteristics such as charge-transfer bands (an exception being organochromium complexes, i.e., complexes bearing Cr-C *σ*-bonds [2–4]), and (d) the paramagnetic nature of chromium(III) (spin 3/2) does not allow studies with NMR techniques [5]. A chromium(III) compound however has been identified as a reactive component of the Glucose Tolerance Factor, GTF [6], where the chromium(III) coordination sphere is occupied by various amino-acids. A biomolecule, termed as low-molecular-weight chromium-binding substance (LMWCr) or chromoduline, is known. It has been isolated from the liver and kidneys of several species. It is an oligopeptide which contains glycine, cysteine, aspartic, and glutamic acid residues [6, 7]. A biomimetic to chromoduline molecule, the trinuclear cation [Cr_3_O(O_2_CCH_2_CH_3_)_6_(H_2_O)_3_]^+^ has been reported [8].

Phenolic acids are examples of phenolic compounds that are present in fruits and plants and constitute a big fraction of the chemical structure of humic substances. The phenolic acids play an important role, probably, in the ability of humic substances to coordinate with the metals, increasing thus their bioavailability [9–11].

**Figure 1:**
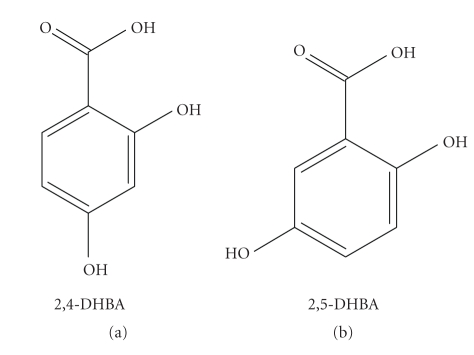


The phenolic acids that were studied for their reactions with chromium(III) and are presented here are 2,4-dihydroxybenzoic acid (2,4-DHBA) and 2,5-dihydroxybenzoic acid (2,5-DHBA) (Figure 1). 

We wish to report, in this work, the kinetics and mechanisms of the reactions between Cr(III) and 2,4- and 2,5-dihydroxybenzoic acids. The two ligands bear both phenolic and carboxylic sites and the study of the mechanisms of their complex formation with chromium(III) as well as the stability of the complexes formed will help in the understanding of the role of complexation in the uptake of chromium(III) by plants.

## 2. Experimental Results

### 2.1. Reagents and Materials

All reagents used were of analytical grade. Dihydroxybenzoic acids (Alfa Aesar) were used as received. They were dissolved in water in concentrations ranging 6.64·10^−3^–4.87·10^−2^ M (2,4-DHBA) and 4.42·10^−3^–4.87·10^−2^ M (2,5-DHBA). In order to avoid transformation and decomposition reactions, the ligand solutions were used shortly after their preparation. Stock solutions of Cr(III) were prepared using Cr(NO_3_)_2_.9H_2_O. Adjustment of the ionic strength was done with KNO_3_. Due to Cr(III) acidic hydrolysis the pH was constantly kept below 4.

### 2.2. Kinetic Experiments

**Figure 2:**
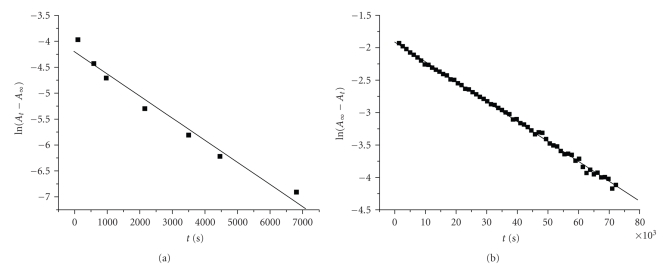
Plots of ln (*A*_*t*_ − *A*_*∞*_) (first stage) and ln (*A*_*∞*_ − *A*_*t*_) (second stage) versus time (a) [2,4-DHBA] = 2,92*·*10^−2^ M, [Cr(III)] = 2,50*·*10^−3^ M, T = 281 K, (b) [2,5-DHBA] = 8,85*·*10^−3^ M, [Cr(III)] = 8,10*·*10^−2^ M, T = 308 K.

The kinetic experiments were carried out at pH values below 4.0 in the presence of air. The kinetics was followed at various wavelengths yielding identical results. The absorbances and the electronic spectra were recorded on a Varian Cary 3E, UV-vis spectrophotometer. Pseudo-first-order conditions were employed for all the experiments. The plots of ln (*A*_*t*_ − *A*_*∞*_) (first stage) and ln (*A*_*∞*_ − *A*_*t*_) (second stage) against time, where *A*_*t*_ and *A*_*∞*_ are absorbances at time *t* and, after the completion of the reaction, were found to be linear for both ligands (Figure 2). The rate constants were calculated from the slope of the lines. At different temperatures for various ligand and chromium(III) concentrations, the experimental results show similar behavior. The uncomplexed Cr(III) species absorbance is not causing a problem in the graphs (Figure 2) since it is included in both *A*_*t*_ and *A*_*∞*_ and is thus eliminated.

The *k*_1_ (first stage) and *k*_2_ (second stage) values at various temperatures are given in Table 1.

Analysis according to activated complex theory gives the activation parameters Δ*Η*_1(2,4)_^≠^, Δ*S*_1(2,4)_^≠^ for 2,4-DHBA/Cr(III), Δ*Η*_1(2,5)_^≠^, Δ*S*_1(2,5)_^≠^ for (2,5-DHBA)/Cr(III), corresponding to *k*_1_ (the first stage of the reaction), calculated from the linear Eyring plots. Activation parameters corresponding to *k*_2_ (the second stage of the reaction for both 2,4- and 2,5-DHBA/Cr(III)) were also calculated from the corresponding linear Eyring plots. All the values of the activation parameters thus obtained are given in Table 2.

The values of *A*_*∞*_ used in Figure 2 and the other graphs were very close to the true values since they were also obtained by plotting *A* = *f*(*t*) at the certain wavelengths. In this way it was possible to check if the reaction was completed. For the reactions studied, *A*_*∞*_ was obtained after more than 3 half-lives.

## 3. Discussion

### 3.1. Kinetics and Mechanisms

Strong hydrogen bonding (intramolecular) occurs in the ligand molecules (2,4- and 2,5-DHBA). Complexation with chromium(III) can occur only after the breaking of these strong bonds. The two ligands exist as neutral molecules (abbreviated as DHBA) and monoanions (abbreviated as DHBA^−^). At pH < 4 chromium(III) exists mainly as hexa-aqua monomeric ion, [Cr(H_2_O)_6_]^3+^ or Cr^3+^, the species [Cr(H_2_O)_5_(OH)]^2+^ or [Cr(OH)]^2+^ being present in small amounts. However, reaction with [Cr(H_2_O)_5_(OH)]^2+^ should be considered over the pH range 3-4 since pK_a_ (Cr^3+^/[Cr(OH)]^2+^) is about 4 [12, 13].

The dissociation equilibria of the two ligands (acids) and the [Cr(H_2_O)_6_]^3+^ complex are

(1) $$\begin{array}{c} \text{DHBA}⇌\text{DHB}{\text{A}}^{-}+{\text{H}}^{+}\;{\text{K}}_{1} \end{array}$$

(2) $$\begin{array}{c} {\text{DHBA}}^{-}⇌{\text{DHBA}}^{2-}+{\text{H}}^{+}\;{\text{K}}_{2} \end{array}$$

(3) $$\begin{array}{c} {\text{DHBA}}^{2-}⇌{\text{DHBA}}^{3-}+{\text{H}}^{+}\;{\text{K}}_{3} \end{array}$$

(4) $$\begin{array}{c} \text{Cr}{\left({\text{H}}_{2}\text{O}\right)}_{6}^{3+}⇌\text{Cr}{\left({\text{H}}_{2}\text{O}\right)}_{5}{\left(\text{OH}\right)}^{2+}+{\text{H}}^{+}\;{\text{K}}_{\text{a}} \end{array}$$

(5) $$\begin{array}{c} \text{PH}<4 \end{array}$$

**Figure 3:**
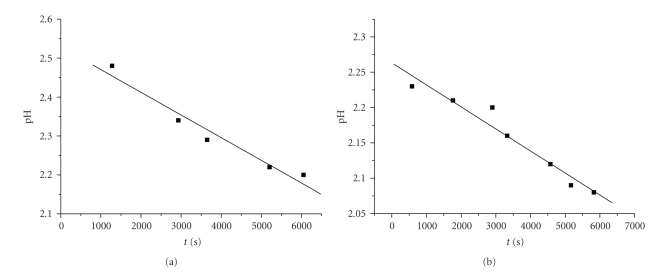
The pH versus time plots of typical mixtures of DHBA/Cr(III). Conditions: (a) [2,4-DHBA]_0_ = 4,87*·*10^−2^ M, [Cr(III)]_0_ = 2,50 10^−3^ M, T = 298 K; (b) [2,5-DHBA]_0_ = 4,87*·*10^−2^ M, [Cr(III)]_0_ = 2,50*·*10^−3^ M, T = 298 K.

The reaction is suggested to be a multistep process since there is a decrease in absorbance at short reaction times and increase at longer reaction times. Because of protonation of the ligands, the groups (hydroxyls and carboxyl) are efficiently blocked and thus the attacks by chromium(III) can take place on them only upon the release of protons, provided that the strong hydrogen bonds have been first broken. This causes a pH decrease in the solution (Figure 3).

The fact that the second first-order step was found to be independent on ligand and chromium(III) concentrations suggests transformations that take place within the first complex that is formed upon the first attack by Cr(III) on the ligand.

In the first step, reaction between the reactive forms of the reactants and chromium(III) takes place, which results in the formation of complex **A**. The first step that is characterized by an absorbance decrease is revealed through experiments conducted at low temperatures. This step is followed by the slower second step, which is independent on both ligand and chromium(III) concentrations.

**Figure 4:**
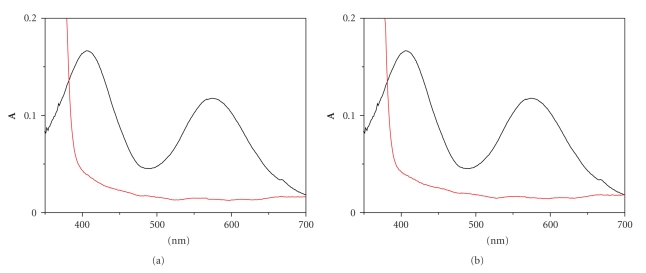
UV-vis spectra of Cr(III)/DHBA solutions: (a) [Cr(III)] = 9.0*·*10^−3^ M and [2,4-DHBA] = 9.0 ^.^10^−3^ M; (b) [Cr(III)] = 9.0*·*10^−3^ M and [2,5-DHBA] = 9.0 ^.^10^−3^ M.

The formation of a light green complex, **C_1_**, from an initially violet (Cr_(aq)_^3+^) and a colourless ligand solution (Figure 4) is observed upon mixing of the reactants. The characterisation of **C_1_**, the light green complex, as an oxygen-bound chromium(III) compound is supported by the UV/vis spectra (*λ*_max_ = 579, 415 nm) and the formation kinetics and subsequent transformation (substitution) kinetics. All of the kinetic data and the associated spectra obtained during the decrease in absorbance are presumed to begin with the already associated complex **C_1_**.

**Figure 5:**
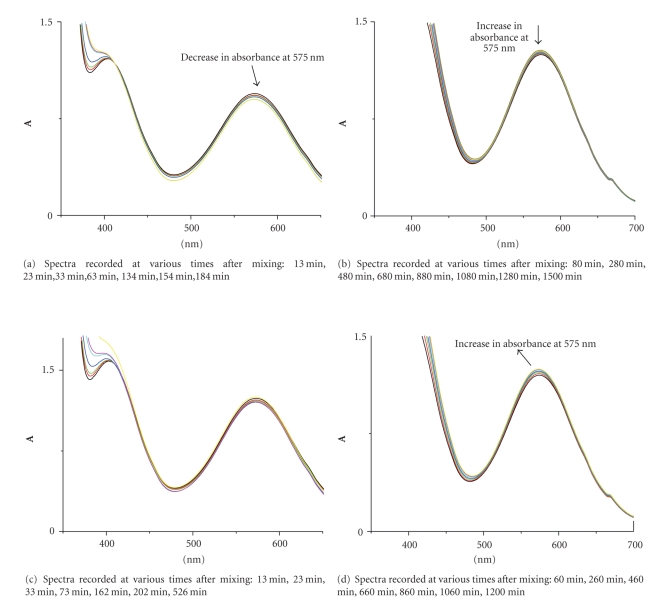
(a) UV-Vis spectra of the reaction mixture Cr(III)/2,4-DHBA. Spectra on the left: [2,4-DHBA] = 8,85*·*10^−3^ M, [Cr(III)] = 0,067 M,T = 288 K. Spectra on the right: [2,4-DHBA] = 8,85*·*10^−3^ M, [Cr(III)] = 0,09 M, T = 298 K. (b) UV-Vis spectra of the reaction mixture Cr(III)/2,5-DHBA. Spectra on the left: [2,5-DHBA] = 8,85*·*10^−3^ M, [Cr(III)] = 0,09 M,T = 288 K. Spectra on the right: [2,5-DHBA] = 8,85*·*10^−3^ M, [Cr(III)] = 0,09 M, T = 298 K.

In Figure 5 spectra of the reaction mixture are recorded at various times after mixing, starting from complex **C_1_**. The final spectrum corresponds to complex **C_2_**. Spectra recorded at intermediate times correspond to mixtures of **C_1 _**and **C_2_**.

#### 3.1.1. First Stage of the Reaction: First Attack of Chromium(III) on the Ligands

The first stage (*k*_1(obs)_) is characterized by an absorbance decrease at 575nm (a d-d transition of Cr_(aq)_^3+^). The attacks of [Cr(H_2_O)_5_OH]^2+^ at the carboxylic ligand groups leading to the complexes **C_1_**, and **C_2_** result in shifting equilibrium (4) to the right. The *k*_1_ pathways occur by attacks of [Cr(H_2_O)_5_OH]^2+^ at the carboxylic ligand groups leading to complexes which in the presence of H^+^ are fast protonated.

**Figure 6:**
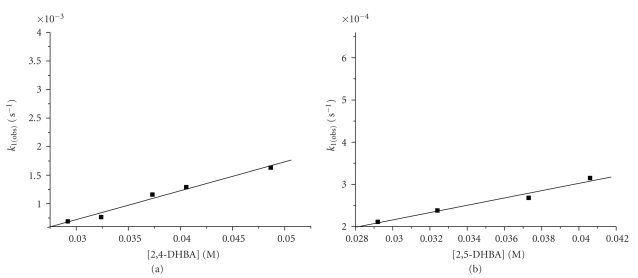
Ligand concentration dependence of *k*_1(obs)_. Conditions: (a) [2,4-DHBA] = 2, 92–4, 87 10^−2^ M, [Cr(III)] = 2, 50 10^−3^ M, T = 296 K, (b) [2,5-DHBA] = 2,92–4,06*·*10^−2^ M, [Cr(III)] = 2,50*·*10^−3^ M, T = 279 K.

In order to obtain information on how many ligands react at the first step, the dependence of *k*_1(obs)_ on the ligand concentration was studied. The kinetics of the first stage of the reaction (*k*_1(obs)_) followed a first-order rate law. The first-order rate constant was found to have a linear dependence on ligand concentration (Figure 6), that is, to obey to the relation *k*_1(obs)_ = *k*_1_[DHBA] + *C*.

Measurements were conducted for the reactions of 2, 4-DHBA with chromium(III) at 281, 287, 290, 296 K and for 2, 5-DHBA with chromium(III) at 279, 283, 291, 298 K.

Below temperature 298 K the plots in Figure 6 are linear within experimental error, and above temperature 298 K the second reaction (*k*_2_) involving the chelation becomes significant. 

In our study of 3,4-dixydroxylphenylpropionic acid with chromium(III) [14] we observed an inverse dependence on [H^+^] which was discussed for the possibility of ligand-H^+^ that is, DHBA^−^ reacting with Cr_aq_^3+^ or DHBA reacting with [Cr(OH)]^2+^.

An I_d_ mechanism is expected for step 1 since the reactive species can only be the conjugate base [Cr(H_2_O)_5_OH]^2+^ for the reactions of which dissociative mechanism I_d_ is supported [15], because of the strong labilizing effect of the coordinated OH^−^ presumably on the trans H_2_O which results to a 10^2^–10^3^-fold enhanced rate for the hydroxy- over the hexa-aqua ion. 

**Scheme 1:**
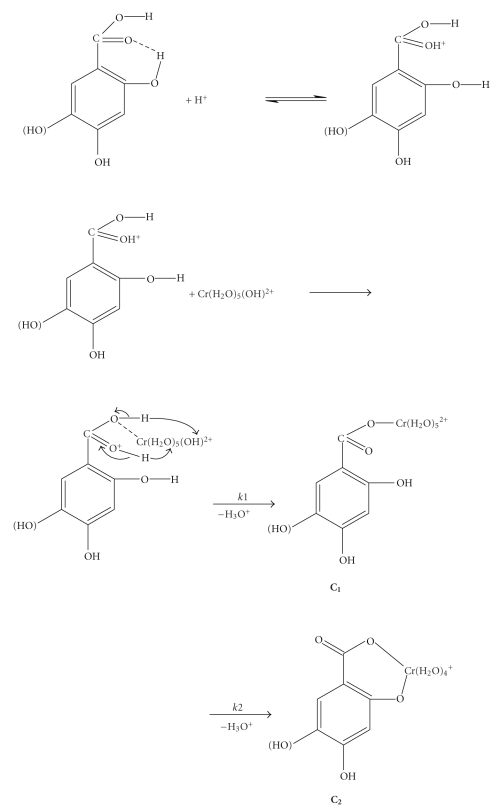
A possible mechanism of the reactions of Chromium(III) with 2,4-and 2,5-dihydroxybenzoic acids in weak acidic aqueous solutions.

Thus, for the first attack, a reaction between [Cr(OH)]^2+^ and the protonated molecule of the ligand can be proposed (Scheme 1), the reaction resulting in proton release (pH decrease). Upon the chelation reaction that follows, proton release takes also place resulting in further pH decrease. Hence for the reactions of 2,4-DHBA and 2,5-DHBA with chromium(III), the mechanism which can be proposed, according to the experimental results (rate laws), is

(6) $$\begin{array}{cc} & {\left[\text{Cr}{\left({\text{H}}_{2}\text{O}\right)}_{6}\right]}^{3+}⇌{\left[\text{Cr}{\left({\text{H}}_{2}\text{O}\right)}_{5}\left(\text{OH}\right)\right]}^{2+}+{\text{H}}^{+}\;{\text{K}}_{\text{a}}\\ & \text{DHBA}+{\text{H}}^{+}⇌{\left[\text{DHBAH}\right]}^{+}\;{\text{K}}_{\text{b}}\\ & {\left[\text{DHBAH}\right]}^{+}+{\left[\text{Cr}{\left({\text{H}}_{2}\text{O}\right)}_{5}\left(\text{OH}\right)\right]}^{2+}\\ & \to {\left[\text{Cr}{\left({\text{H}}_{2}\text{O}\right)}_{5}\left(\text{OH}\right)\left({\text{DHBAH}}^{+}\right)\right]}^{3+}\overset{k_{1}}{\to }\\ & \to {\left[\text{Cr}{\left({\text{H}}_{2}\text{O}\right)}_{5}\left({\text{DHBA}}^{-}\right)\right]}^{2+}+{\text{H}}_{3}\text{O}\\ & {\left[\text{Cr}{\left({\text{H}}_{2}\text{O}\right)}_{5}\left({\text{DHBA}}^{-}\right)\right]}^{2+}\overset{k_{2}}{\to }{\left[\text{Cr}{\left({\text{H}}_{2}\text{O}\right)}_{4}\left({\text{DHBA}}^{2-}\right)\right]}^{+}+{\text{H}}_{3}{\text{O}}^{+}\\ & {\text{K}}_{\text{a}}=\left[\text{Cr}{\left({\text{H}}_{2}\text{O}\right)}_{5}{\left(\text{OH}\right)}_{}^{2+}\right]\;\left[{\text{H}}^{+}\right]/\left[\text{Cr}{\left({\text{H}}_{2}\text{O}\right)}_{6}^{3+}\right]\\ & {\text{K}}_{\text{b}}=\left[{\text{DHBAH}}^{+}\right]/\left[\text{DHBA}\right]\left[{\text{H}}^{+}\right] \end{array}$$

Hence, R = *k*_1_ [DHBAH^+^] [Cr(H_2_O)_5_(OH)^2+^] and by substituting [Cr(H_2_O)_5_(OH)^2+^] and [DHBAH^+^], results the following:

R = *k*_1_K_b_ [DHBA] [H^+^] K_a_ [Cr(H_2_O)_6_^3+^] / [H^+^] = *k*_1_K_b_K_a_ [DHBA] [Cr(H_2_O)_6_^3+^]

and *k*_1(obs)_ = *k*_1_K_b_K_a_ [DHBA] (in excess DHBA).

The *k*_2(obs)_ dependence on chromium(III) and ligand concentration were studied in order to find if another Cr(III) ion and / or ligand molecule is entering the compound C_1 _ which is produced in step 1.

**Figure 7:**
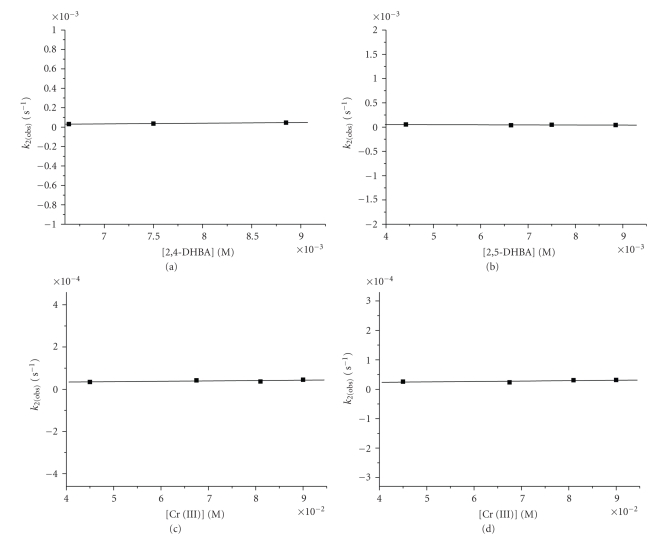
Ligand and Cr(III) concentration dependence of *k*_2(obs)_. Conditions: (a) [2,4-DHBA] = (6.64–8.85) 10^−3^ M, [Cr(III)] = 1.25 10^−3^ M, T = 313 K, (b) [2,5-DHBA] = (4,42–8.85) 10^−3^ M, [Cr(III)] = 1.25 10^−3^ M, T = 313 K, (c) [2,4-DHBA] = 8.8510^−3^ M, [Cr(III)] = (4.5–9.00) 10^−2^ M, T = 313 K, (d) [2,5-DHBA] = 8.85 10^−3^ M, [Cr(III)] = (4.5–9.0) 10^−2^ M, T = 308 K.

The concentrations of DHBA and of Cr(III) have no effect on the observed rate constants *k*_2(obs)_ in the range of concentrations applied as is shown in Figure 7. Higher concentrations were not possible to be achieved due to solubility reasons of 2,4- and 2,5-DHBA in weak acidic aqueous solutions. The *k*_2(obs)_ values, for the DHBA and chromium(III) concentration range studied, at various temperatures are given in Table 1 and the corresponding ΔH_2(obs)_^≠^, ΔS_2(obs)_^≠^ values in Table 2.

The activation parameters are deduced from the temperature dependence experiments and lead to structures of the activated complexes and of the type of mechanism taking place. The negative values of ΔS^≠^ (ΔS_1(obs)_^≠^) suggest an associative mechanism for the first stage of the reaction. [Cr(H_2_O)_5_OH]^2+^, however, as stated above, reacts/follows an I_d_ mechanism. This suggests that composite activation parameters Δ*Η*_1(obs)_^≠^ = Δ*Η*_0_ + Δ*Η*_1_^≠^ and ΔS_1(obs)_^≠^ = ΔS_0_ + ΔS_1_^≠^ are applied where ΔH_0_ and ΔS_0_ correspond to a pre-equilibrium K_0_ and ΔH_1_^≠^ and ΔS_1_^≠^ correspond to the first step (complexation, *k*_1_).

Thus the resulting negative values of ΔS^≠^ as well as the resulting values of ΔH^≠^ do not correspond to step 1, which is taking place by an I_d_ mechanism due to the reactive species [Cr(H_2_O)_5_OH]^2+^.

The negative values of ΔS_2(obs)_^≠^, the independence on ligand(s) and chromium(III) concentrations of *k*_2(obs)_, the increase of the extinction coefficients (increase in absorbance), and the pH decrease (Figure 3) led to the assignment of the studied transformations as associatively activated substitution reactions of water molecules from the Cr(III) coordination sphere by the ligand(s) through chelation, with concomitant proton release (Scheme 1).

The absorbance increase of the second stage is due to the chelation and the pH decrease is due to the release of protons upon the course of these reactions (Figure 3, Scheme 1). These changes are attributed to the formation of an oxygen-bound chromium(III)-DHBA acid complex (*k*_1_) followed by chelation (*k*_2_).

Stoichiometry of 1 : 1 for the reaction of Cr(III) with 2,4- and 2,5-DHBA is proposed, according to the observed *k*_1_ dependence on ligand concentration and the chelation reactions in the Cr(III) center. In accordance with the stoichiometry resulting from the kinetics are the elemental analysis results for the isolated in the solid form final product. The isolation was achieved by addition of KOH solution in the final reaction mixture. Thus the experimental percentages C = 11.82% and H = 3.49% correspond to the formula [Cr(2,5-DHBA_−3H_)(H_2_O)_4_]^.^3KNO_3_^.^8H_2_O (MW = 722) for which the calculated percentages (theoretical values) for C and H are C = 11.63% and H = 3.74%.

Hence, the formation of **C_2_**, the final chelated complex, occurs actually in at least two stages. 

#### 3.1.2. Structures of the Activated Complexes *C*_1_^≠^ and *C*_2_^≠^

**Scheme 2:**
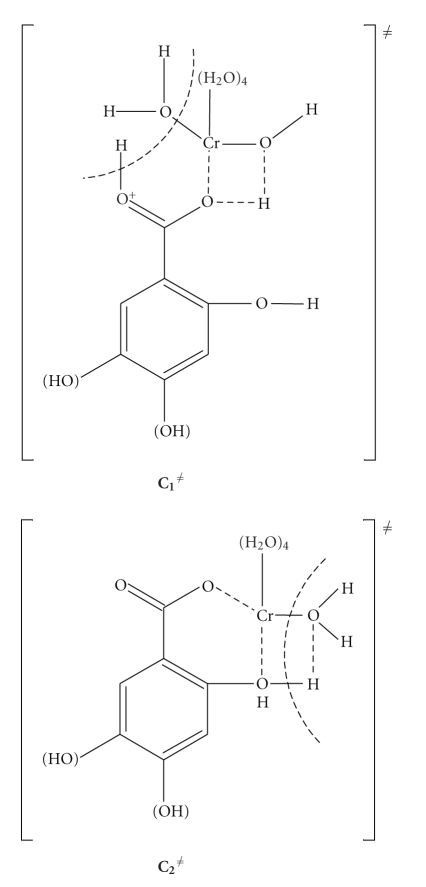
Activated complexes **C**_1_^≠^ and **C**_2_^≠^.

These are given in Scheme 2. Associative mechanisms have been found to be operative in reactions of Cr(III) [4, 16, 17]. The negative values of the entropies of activation Δ*S* ^≠^ that have been calculated for our systems suggest an associative mode of activation. The original complex **C_1_** could also be a chelate itself. This could easily explain the formation of compounds containing Cr-O bonds. The negative entropies of activation Δ*S* ^≠^, however, suggest the formation of more structured transition states from the less organized reactants. Therefore, complex **C_1_** is not in chelated form and the mechanisms shown in Scheme 1 are thus supported. If the *k*_2_ path proceed via attack by an external ligand on the complex **C_1_**, the *k*_2_ path could be ligand concentration dependent. In the suggested mechanism the phenolic groups act as internal attacking groups to the chromium-bound H_2_O molecules and supply a proton to the H_2_O in the same complex (Scheme 2), which is released as H_3_O^+^.

The final product **C_2_** could be chelated at the carboxylic group through its oxygens. This alternative is not supported because a five-membered ring, which is formed according to the suggested mechanism, is more stable than the four-membered ring which would result if chelation at the carboxylic group took place.

The UV/vis spectrum which exhibits a maximum at 575 nm and a shoulder at 412 nm (d→d transitions of Cr(III)) upon complexation changes, as expected, due to continuous changes in the ligand field. This is caused by the new ligand(s) which enters in the coordination sphere of Cr(III). Water molecules are being replaced by the ligand groups leading to **C_1_** and finally to **C_2_**. The conditions are kept acidic suggesting that oxidation does not take place and the only changes that are expected in the spectrum are only those being caused by the change of the ligand field. This means that only a shift of the maximum and disappearance of the shoulder in the UV region take place; this last change is due to the high absorptivities of the resulting complexes that hide the shoulder.

The chelation of the first formed complex produces PhO-bound chromium(III) species, characterized by the UV/vis spectra and the kinetic behavior which are typical of other known complexes containing Cr-O bond(s) [16, 17].

### 3.2. Structure of the Complexes-Mode of Binding

The isolation of a pure product **C_2_** is already mentioned (page 18) that corresponds to the formula [Cr(2,5-DHBA_−3H_)(H_2_O)_4_]^.^3KNO_3_^.^8H_2_O according to the elemental analysis and all other experimental data that suggest, as previously discussed, that binding is taking place through the carboxylic and the nearby phenolic group (Scheme 1, for ligands 2,4- and 2,5-DHBA). Catecholic type of binding is established in the coordination complexes of dihydrocaffeic, caffeic and ferulic acids with Co(II), Ni(II), Cu(II), Fe(III), Mn(II), Mn(III), V(V), V(IV,V), Zn(II) [18–22], and dihydroxyphenylpropionic acid (dihydrocaffeic acid) with Cr(III) [14]. The same type of coordination, that is, catecholic, has been also suggested for the reaction of caffeic acid with Cr(III) [23] and 3,4-dihydroxybenzoic acid with Cr(III) [24]. In some of the above presented structures both catecholic and carboxylic binding is observed. Catecholic type of coordination was found in Fe(III)-2,3-dihydroxybenzoic acid complex for which the kinetics of the coordination and oxidation process have been reported [25, 26]. In the systems studied in this work (2,4- and 2,5-dihydroxybenzoic acids and chromium(III)), binding is supported to take place between chromium(III) and the carboxylic and the 2-phenolic group of the ligands. Analogous binding between the carboxylic and the 2-phenolic group of the ligand was reported for the reaction between chromium(III) and 2,3-dihydroxybenzoic acid [27].

## 4. Conclusions

Our results indicate that the reactions between chromium(III) and 2,4- and 2,5-DHBA in weak acidic aqueous solutions follow a two-step mechanism according to which an initial step 1 consisting of an attack between the acid molecule (ligand) and the [Cr(H_2_O)_5_OH]^2+^ complex to give a carboxylate bound Cr(III) is followed by a nonligand and nonchromium(III)-dependent step assigned to be a chelation step due to the negative entropies of activation. Both steps are followed by pH decrease suggesting that proton release is taking place. The protons are released in an associatively activated mode.

The first step depends on 2,4- or 2,5-DHBA concentration and [Cr(H_2_O)_5_OH]^2+^ is the reactive metal complex. This is followed by a slower step, the rate of which depends only on the concentrations of their respective intermediate complexes **C_1_**, with negative entropies of activation, suggesting that the transition states are associatively activated.

The negative values of the entropies of activation of the second step, the independence on ligand and chromium(III) concentrations of their rate, the displacement of the wavelengths maxima, the increase of the extinction coefficients, the pH decrease due to release of protons upon complexation, and the various transformations lead to the mechanism presented in Scheme 1. The observed transformations are assigned as substitution of water molecules from the coordination sphere of Cr(III) by the ligand through complexation followed by chelation. A dissociative I_*d*_ mechanism is supported for step 1 and an associative mechanism for step 2.

## Figures and Tables

**Table 1:** Values of *k*_1_ and *k*_2_ at various temperatures.

2,4-DHBA			2,5-DHBA		
T(K)	*k*_1_(M^−1^s^−1^) (·10^+2^)	*k*_2_ (s^−1^) (·10^+5^)	T(K)	*k*_1_(M^−1^s^−1^) (·10^+2^)	*k*_2_ (s^−1^) (·10^+5^)
281	1.57		279	0.87	
287	2.10		283	1.24	
290	2.14		291	2.12	
296	5.06		298	4.17	
298		1.45	298		1.40
303		1.86	303		1.89
308		2.88	308		2.78
313		3.95	313		4.44
313		3.48	313		4.31

**Table 2:** Activation parameters for steps 1 (*k*_1_) and 2 (*k*_2_) for the reactions of the two ligands 2,4- and 2,5-DHBA with chromium(III).

	2,4-DHBA	2,5-DHBA
Δ*Η*_1_^≠^(kJ/mol^−1^)	49,5	60,3
Δ*S*_1_^≠^(J mol^−1^K^−1^)	−103,7	−68,0
Δ*Η*_2_^≠^ (kJ/mol^−1^)	45,13	54,55
ΔS_2_^≠^ (J mol^−1^K^−1^)	−185,9	−154,8

## References

[1] Ochiai E-I (1987). *General Principles of Biochemistry of the Elements in Biochemistry of the Elements*.

[2] Petrou A, Vrachnou-Astra E, Katakis D (1980). A new series of organochromium complexes formed in aqueous solutions. *Inorganica Chimica Acta*.

[3] Petrou A, Vrachnou-Astra E, Konstantatos J, Katsaros N, Katakis D (1981). Kinetics and mechanisms of aquation of some *σ*-bonded organochromium complexes. *Inorganic Chemistry*.

[4] Petrou AL (1993). Kinetics and mechanism of the reaction between chromium(II) and 1,2-bis(2-pyridyl)ethylene in acidic aqueous solutions. *Journal of the Chemical Society, Dalton Transactions*.

[5] Vincent JB (2003). Recent advances in the biochemistry of chromium(III). *Journal of Trace Elements in Experimental Medicine*.

[6] Yamamoto A, Wada O, Ono T (1987). Isolation of a biologically active low-molecular-mass chromium compound from rabbit liver. *European Journal of Biochemistry*.

[7] Davis CM, Vincent JB (1997). Isolation and characterization of a biologically active chromium oligopeptide from bovine liver. *Archives of Biochemistry and Biophysics*.

[8] Michele Davis C, Royer AC, Vincent JB (1997). Synthetic multinuclear chromium assembly activates insulin receptor kinase activity: functional model for low-molecular-weight chromium-binding substance. *Inorganic Chemistry*.

[9] Cockerham G, Shane BS (1994). *Basic Environmental Toxicology*.

[10] Dudarchik VM, Smychnik TP, Terentyev AA (1998). *XIXth International Conference on Polyphenols*.

[11] Lehtonen T, Peuravuori J, Pihlaja K (2004). Degradative analysis of aquatic fulvic acid: CuO oxidation versus pyrolysis after tetramethylammonium hydroxide treatments in air and helium atmospheres. *Analytica Chimica Acta*.

[12] Baes CF, Mesner RE (1976). *The Hydrolysis of Cations*.

[13] Cotton FA, Wilkinson G, Murillo CA, Bochmann M (1999). *Advanced Inorganic Chemistry*.

[14] Petrou AL, Paraskevopoulou P, Chrysikopoulou M (2004). Kinetics and mechanism of the reaction between chromium(III) and 3,4-dihydroxyphenylpropionic (dihydrocaffeic) acid in weak acidic aqueous solutions. *Journal of Inorganic Biochemistry*.

[15] Wilkins RF (1991). *Kinetics and Mechanisms of Reactions of Transition Metal Complexes*.

[16] Swaddle TW, Stranks DR (1972). Mechanistic information from pressure and temperature effects on the rate of transfer of oxygen-18 from aquopentaamminechromium(III) and aquopentaamminerhodium(III) ions to solvent water. *Journal of the American Chemical Society*.

[17] Ramasami T, Sykes AG (1976). Mechanistic implications of kinetic data for the formation and aquation of acidopentaamminechromium(III) complexes, Cr(NH_3_)_5_X^2+^, X^−^ = NCS^−^, CCl_3_CO_2_
^−^, CF_3_CO_2_
^−^, Cl^−^, Br^−^, and I^−^. Evidence for a dissociative mechanism. *Inorganic Chemistry*.

[18] Petrou AL, Koromantzou MV, Tsangaris JM (1991). Coordination complexes of 3,4-dihydroxyphenylpropionic acid (dihydrocaffeic acid) with copper(II), nickel(II), cobalt(II) and iron(III). *Transition Metal Chemistry*.

[19] Petrou AL (1993). Binuclear vanadium(V) and vanadium(IV, V) complexes of dihydrocaffeic, caffeic and ferulic acids. *Transition Metal Chemistry*.

[20] Petrou AL, Perlepes SP (1994). Preparation and properties of manganese(II) and manganese(III) complexes possessing ligands with carboxylate and phenolic/phenoxide groups. *Chimika Chronika, New Series*.

[21] Petrou AL, Perlepes SP (1995). Oligonuclear zinc(II) complexes of dianion of hydrocaffeic, caffeic and ferulic acids. *Chimika Chronika, New Series*.

[22] Petrou AL, Koromantzou MV, Tsangaris JM (1993). Coordination complexes of caffeic and ferulic acids with Cu(II), Ni(II), Co(II) and Fe(III). *Chimika Chronika, New Series*.

[23] Thoma V, Tampouris K, Petrou AL (2008). Kinetics and mechanism of the reaction between chromium(III) and 3,4-dihydroxy-phenyl-propenoic acid (caffeic acid) in weak acidic aqueous solutions. *Bioinorganic Chemistry and Applications*.

[24] Zavitsanos K, Tampouris K, Petrou AL (2008). Reaction of chromium(III) with 3,4-Dihydroxybenzoic acid: kinetics and mechanism in weak acidic aqueous solutions. *Bioinorganic Chemistry and Applications*.

[25] Xu J, Jordan RB (1988). Equilibrium and kinetic studies of the complexing of iron(III) by 1,2-dihydroxybenzene derivatives. *Inorganic Chemistry*.

[26] Xu J, Jordan RB (1988). Kinetics and mechanism of the oxidation of 2,3-dihydroxybenzoic acid by iron(III). *Inorganic Chemistry*.

[27] Petrou AL, Thoma V, Tampouris K (2010). Kinetics and mechanism of the reaction chromium(III) and 2,3-dihydroxybenzoic acid in weak acidic aqueous solutions. *Bioinorganic Chemistry and Applications*.

